# Cell Membrane Remodeling Mediates Polymyxin B Resistance in *Klebsiella pneumoniae*: An Integrated Proteomics and Metabolomics Study

**DOI:** 10.3389/fmicb.2022.810403

**Published:** 2022-02-10

**Authors:** Xinyi Chen, Jingjing Tian, Can Luo, Xiaofan Wang, Xianping Li, Min Wang

**Affiliations:** Department of Laboratory Medicine, The Second Xiangya Hospital of Central South University, Changsha, China

**Keywords:** proteomics, metabolomics, *Klebsiella pneumoniae*, polymyxin B resistance, membrane remodeling

## Abstract

Polymyxin B (PB) is introduced into the clinic as the last-line therapy against carbapenem-resistant *Klebsiella pneumoniae* (CRKP). Unfortunately, increased resistance to PB in *Klebsiella pneumoniae* (*K. pneumoniae*) has threatened global health. Resistance of *K. pneumoniae* to PB was induced by passaging in serial concentrations of PB and determined by microbroth dilution method. Growth characteristics of induced strains including growth curve, reversibility of resistance, and biofilm formation (crystal violet staining method) were measured. This study employed TMT-labeled quantitative proteomics and LC-MS/MS metabolomics analysis to investigate the key biological processes associated with PB resistance in *K. pneumoniae*. A total of 315 differentially expressed proteins (DEPs) were identified, of which 133 were upregulated and 182 were downregulated in the PB-resistant *K. pneumoniae*. KEGG enrichment analysis revealed that the DEPs were mainly involved in ATP-binding cassette (ABC) transporters and cationic antimicrobial peptide (CAMP) resistance. Proteins related to central carbon metabolism were inhibited in the PB-resistant *K. pneumoniae*, but proteins mediating LPS modification were activated. Transcriptional levels of CAMP resistance-related proteins were significantly different between PB-susceptible and -resistant *K. pneumoniae*. PB treatment led to an increase in reactive oxygen species (ROS) levels of *K. pneumoniae*. Metabolomics data demonstrated that 23 metabolites were significantly upregulated in PB-resistant *K. pneumoniae* and 5 were downregulated. The differential metabolites were mainly lipids, including glycerophospholipids, sphingolipids, and fatty acids. Exposure to PB resulted in increased level of phospholipid transport gene *mlaF* in *K. pneumoniae*. Our study suggested that membrane remodeling and inhibited central carbon metabolism are conducive to the development of PB resistance in *K. pneumoniae*.

## Introduction

*Klebsiella. pneumoniae* is the main cause of hospital-acquired infection, including urinary tract infections, pneumonia, and bacteremia (Kontopidou et al., 2014; Balkhair et al., 2019; Marques et al., 2019). Infection caused by carbapenem-resistant *Enterobacteriaceae* (CRE) has become a public health problem due to long hospital stays, high clinical failure rates, and high cost (Kontopidou et al., 2014). The World Health Organization (WHO) has emphasized CRE, including CRKP, as one of the three top-priority pathogens urgently in need of new therapies (Tacconelli et al., 2017).

Options for treating CRKP infections are extremely limited. Polymyxin B and E (also called colistin), a family of cyclic lipopeptides, are considered as the last-resort therapeutic options against Gram-negative “superbugs,” particularly for CRKP (Aye et al., 2020). The bactericidal activity of polymyxin is mainly achieved by electrostatic interaction between the cationic L-α, γ- diaminobutyric acid (Dab) residues of the polymyxin molecule and anionic phosphate groups on the lipid A section of lipopolysaccharide (LPS) in the outer membrane (OM) (Velkov et al., 2010). This interaction will cause insertion of PB molecule into the fatty acyl layer of the OM and damage of LPS (Velkov et al., 2010; Poirel et al., 2017). As a result, permeability of the bacterial cell membrane increased, eventually leading to cell death (Falagas and Kasiakou, 2005; Poirel et al., 2017). Alarmingly, resistance to polymyxin in CRE has been increasingly observed due to the growing use of polymyxin, with polymyxin-resistant CRKP accounting for the majority (Macesic et al., 2020).

At present, the main mechanism of PB resistance in Gram-negative bacteria is LPS modification. Most commonly, polymyxin resistance in Gram-negative bacteria occurs via modifications of lipid A with positively charged moieties, including 4-amino-4-deoxy-L-arabinose (L-Ara4N) and phosphoethanolamine (pEtN). Such modifications of lipid A could diminish the electrostatic affinity with polymyxin, reducing attachment of polymyxin to the external membrane of bacteria (Aye et al., 2020). In *Enterobacteriaceae*, many genes regulated by the two-component system (TCS) PhoP/PhoQ and PmrA/PmrB can confer polymyxin resistance, and PhoP/PhoQ is able to activate PmrAB TCS through the connector PmrD (Chen et al., 2021). In *K. pneumoniae*, addition of L-Ara4N is achieved by mutations in PhoP/PhoQ and PmrA/PmrB TCS and the following constitutive expression of the *arnBCADTEF* operon (Sun et al., 2020). The pEtN transferase could be encoded by either *mcr* or *eptA* genes (Nang et al., 2019; Liu Y. et al., 2020). More problematically, the plasmid-mediated polymyxin resistance via *mcr-1* and its variants has been noted since 2015. The emergence of *mcr* genes indicates that polymyxin resistance may readily spread (Liu Y. et al., 2020). Thus, there is an urgent need to address the exact mechanisms responsible for this resistance.

To date, systems biology has provided an opportunity to explain the specific mechanisms of antibiotic activity and resistance (Han et al., 2019a,b; Aye et al., 2020; Sun et al., 2020). In the present study, metabolomics and proteomics analysis were performed on PB susceptible and resistant *K. pneumoniae* to demonstrate metabolic and proteomic perturbations associated with PB resistance. Integrated omics showed that membrane remodeling, suppressed tricarboxylic acid (TCA) cycle, and glycolysis/gluconeogenesis occurred in PB-resistant *K. pneumoniae*, suggesting that these changes may contribute to the development of PB resistance.

## Results

### Induction of Polymyxin B Resistance and Changes in Growth Characteristics of *Klebsiella pneumoniae*

**FIGURE 1:**
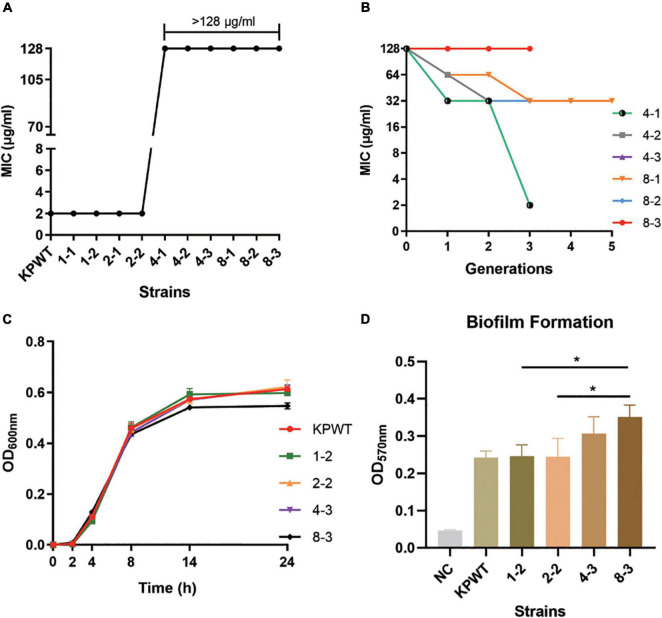
Resistance to polymyxin B (PB) and growth characteristics of *Klebsiella pneumoniae* parent isolate and induced strains. **(A)** PB MIC against *K. pneumoniae* strains before and after induction. **(B)** Changes in PB MICs of induced strains passaged in antibiotic-free medium. **(C)** Growth curve of parent isolate and induced strains. **(D)** Comparison of biofilm formation ability. The data shows the mean ± SD of three independent replicates. *P < 0.05. NC, negative control. The induced strains were named in a PB concentration-passage manner, e.g., 1-2, the second generation of strain grown in the medium with PB concentration of 1 μg/ml.

Ten PB susceptible CRKP clinical isolates were collected from neonatal sputum, gastric juice, and feces. Resistance was induced by experimental evolution in increasing concentrations of PB as described in Methods. After induction, these strains were successfully resistant to PB, with MIC value ≥ 4 μg/ml. Among these ten CRKP isolates, strain KPWT showed the strongest PB resistance after induction, so it was selected for subsequent studies. Compared with the parent isolate, MIC to PB of the induced resistant strains increased significantly to > 128 μg/ml (> 16-fold) (Figure 1A). The descendant strains had slight fluctuations in the MICs of imipenem, meropenem, TZP, nitrofurantoin, levofloxacin, and tigecycline (Table 1).

**TABLE 1:** Antimicrobial susceptibility of KPWT and its induced descendant strains.

Antibiotics	MIC of strains (μg/ml)				
	KPWT	1–2	2–2	4–3	8–3
AMK	≤ 8	≤8	≤8	≤ 8	≤8
GEN	≤ 2	≤2	≤ 2	≤2	≤ 2
TOB	≤2	≤ 2	≤2	≤ 2	≤2
ETP	** > 2**	** > 2**	** > 2**	** > 2**	** > 2**
IPM	**4**	2	2	2	2
MEM	**8**	**4**	**4**	**4**	**4**
CZ	** > 16**	** > 16**	** > 16**	** > 16**	** > 16**
CXM	** > 16**	** > 16**	** > 16**	** > 16**	** > 16**
FOX	** > 16**	** > 16**	** > 16**	** > 16**	** > 16**
CAZ	** > 32**	** > 32**	** > 32**	** > 32**	** > 32**
CRO	** > 32**	** > 32**	** > 32**	** > 32**	** > 32**
FEP	** > 16**	** > 16**	** > 16**	** > 16**	** > 16**
SCP	> 32/8	> 32/8	> 32/8	> 32/8	> 32/8
AZT	** > 32**	** > 32**	** > 32**	** > 32**	** > 32**
AMC	**32/16**	**32/16**	**32/16**	**32/16**	**32/16**
SAM	> 16/8	> 16/8	> 16/8	> 16/8	> 16/8
TZP	32/4	16/4	16/4	16/4	32/4
COL	≤1	≤ 1	≤1	** > 4**	** > 4**
SXT	≤1/19	≤ 1/19	≤1/19	≤ 1/19	≤1/19
C	≤4	≤ 4	≤4	≤ 4	≤4
FF	≤16	≤ 16	≤16	≤ 16	≤16
FM	32	64	64	** > 64**	** > 64**
CIP	≤0.5	≤ 0.5	≤0.5	≤ 0.5	≤0.5
LVX	≤1	≤ 1	≤1	≤ 1	**2**
MXF	≤0.5	≤ 0.5	≤0.5	≤ 0.5	≤0.5
NOR	≤2	≤ 2	≤2	≤ 2	≤2
MINO	2	2	2	4	4
TET	≤2	≤ 2	≤2	≤ 2	≤2
TGC	≤1	**4**	2	2	2

*AMK, amikacin; GEN, gentamicin; TOB, tobramyxin; ETP, ertapenem; IPM, imipenem; MEM, meropenem; CZ, cefazolin; CXM, cefuroxime; FOX, cefoxitin; CAZ, ceftazidime; CRO, ceftriaxone; FEP, cefepime; SCP, cefoperazone-sulbactam; AZT, aztreonam; AMC, amoxicillin-clavulanate; SAM, ampicillin-sulbactam; TZP, piperacillin-tazobactam; COL, colistin; SXT, trimethoprim-sulfamethoxazole; C, chloramphenicol; FF, Fosfomycin w/G6P; FM, nitrofurantoin; CIP, ciprofloxacin; LVX, levofloxacin; MXF, moxifloxacin; NOR, norfloxacin; MI, minocycline; TET, tetracycline; TGC, tigecycline. Resistance is emphasized in bold.*

The induced resistance of *K. pneumoniae* by 8 μg/ml PB remained stable after 3-5 passages in an antibiotic-free growth medium (Figure 1B). However, the resistance induced by 4 μg/ml PB was less stable, and these strains restored PB sensitivity within three passages without antibiotic. Although strain 8-3 showed a minor fitness loss compared with the parental strain, growth rate of the induced strains was basically the same as that of the parental strain (Figure 1C). Biofilm formation capacities of resistant strains were also gradually increased during induction (Figure 1D).

The parent isolate KPWT and its descendant strain 8-3 were prepared for subsequent proteomics and metabolomics studies.

### Overview of Proteome Alterations Between Polymyxin B Susceptible and Resistant *Klebsiella pneumoniae*

**FIGURE 2:**
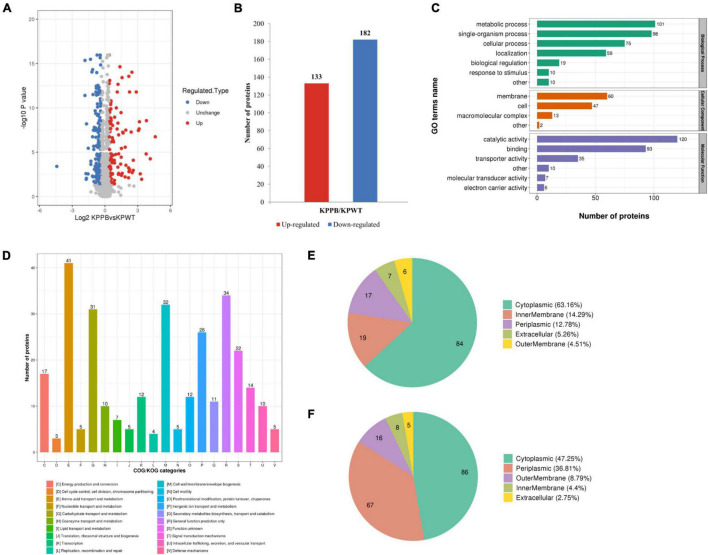
Quantitative proteomics analysis of polymyxin B (PB) resistant and susceptible *Klebsiella pneumoniae*. **(A)** Volcano plot of the differentially expressed proteins (DEPs). The *x*-axis represents expression changes and the *y* axis represents the statistical significance between the two groups. **(B)** Numbers of increased and decreased DEPs. **(C)** Number of DEPs in the biological process, cellular component, and molecular function categories revealed by GO annotation. **(D)** Functional classification of the DEPs in COG categories. Subcellular localization of upregulated **(E)** and downregulated **(F)** DEPs in PB-resistant *K. pneumoniae* compared to –susceptible *K. pneumoniae*.

In the current study, a total of 3352 proteins were identified in each sample using the TMT-labeled quantitative proteomics. The proteomics data showed that the PB-susceptible and -resistant *K. pneumoniae* demonstrated different expression patterns of proteins (details in Supplementary Data Sheet 1). 315 DEPs were screened [KPPB vs. KPWT, fold-change (FC) > 1.3 or < 0.77, *t*-test, *P* < 0.05], of which 182 were found downregulated and 133 were upregulated in the PB-resistant *K. pneumoniae* (Figures 2A,B).

### Functional Classification and Enrichment Analysis of Differentially Expressed Proteins in Polymyxin B Resistant and Susceptible *Klebsiella pneumoniae*

The effects of DEPs on cellular processes were clarified using Gene Ontology (GO) annotation and COG (Clusters of orthologous groups of proteins) analysis. Subcellular locations of DEPs were then predicted by WoLF PSORT: protein localization predictor (Horton et al., 2007).

The GO annotation explained various biological processes impacted by DEPs from three aspects: biological process, cellular component, and molecular function (Figure 2C). The biological processes associated with DEPs were as follows: 101 proteins were associated with metabolic process, 98 proteins with single organic process, and 75 with cellular process. Localization of DEPs in cellular components was mainly concentrated in membrane (60 proteins), cell (47proteins) and macromolecular complex (13 proteins). The number of DEPs involved in the top three items of molecular function was as follows: 120 proteins were associated with catalytic activity, 93 proteins with binding, and 35 proteins with transporter activity.

Then functional classification of DEPs was carried out with COG analysis (Figure 2D): 41 proteins were associated with amino acid transport and metabolism, 34 proteins with general function prediction only, and 32 proteins with cell wall/membrane/envelope biogenesis. Another category with more than 30 DEPs was carbohydrate transport and metabolism (31 proteins).

Predicted subcellular locations of the DEPs are shown in a pie chart. Among the upregulated DEPs, 84 proteins were localized in cytoplasmic (63.16%), 19 proteins in inner membrane (14.29%), and 17 proteins in periplasm (12.78%) (Figure 2E). For the downregulated DEPs, 86 proteins were localized in cytoplasm (47.25%), 67 proteins in periplasmic (36.81%) and 16 proteins in outer membrane (8.79%) (Figure 2F). Taken together, these results indicated that the physiological activities were widely affected in cytoplasm of PB-resistant *K. pneumoniae*, but were inhibited in periplasm.

**FIGURE 3:**
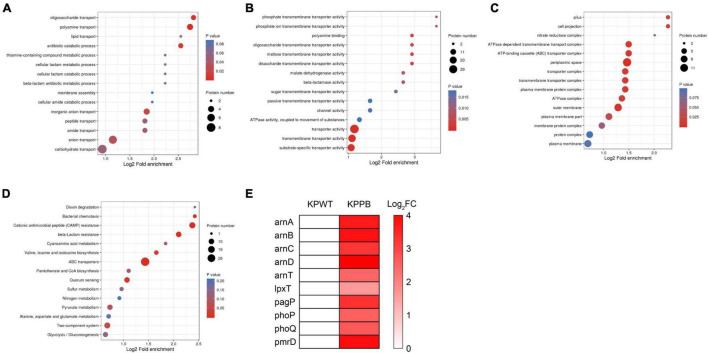
Enrichment analysis of the differentially expressed proteins (DEPs) between polymyxin B (PB) resistant and susceptible *Klebsiella pneumoniae*. GO functional enrichment analysis of biological process **(A)**, molecular function **(B),** and cellular component **(C)**. **(D)** KEGG enrichment plot demonstrated the top 15 pathways of the DEPs. **(E)** Expression pattern of the DEPs associated with LPS modification.

Fisher’s exact test was used to further identify significantly enriched GO classification and protein domains of the DEPs by *P*-value. Results indicated that biological processes of DEPs were significantly enriched in polyamine transport, oligosaccharide transport, and antibiotic catabolic transport (*P* < *0.05*) (Figure 3A). Molecular function of the DEPs was concentrated on transporter activity, transmembrane transporter activity, and substrate-specific transporter activity (Figure 3B). Furthermore, DEPs were also significantly enriched in disaccharide transmembrane transport activity, maltose transmembrane transport activity, oligosaccharide transmembrane transport activity, and ATPase complex, suggesting that ATP production may be affected in the PB-resistant *K. pneumoniae*. For cellular component, the DEPs were markedly enriched in the periplasmic space, outer membrane, and ATP-binding cassette (ABC) transporter complex (Figure 3C). Taken together, the transport process and activity of *K. pneumoniae* was considered to have a potential effect on PB resistance.

### Multiple Biological Pathways Were Affected in PB-resistant *K. pneumoniae.*

To reveal the underlying molecular mechanisms associated with PB resistance, the DEPs were then submitted to perform pathway enrichment analysis based on KEGG database (Figure 3D). A majority of the DEPs were involved in ABC transporters, cationic antimicrobial peptide (CAMP) resistance, beta-lactam resistance, bacterial chemotaxis, valine, leucine, and isoleucine biosynthesis, and quorum sensing pathways (*P* < 0.05).

Diverse LPS modifications were activated in PB-resistant *K. pneumoniae*. The two-component system PhoP (5.5-fold) and PhoQ (6.0-fold) with its downstream PmrD (13.7-fold), ArnC (8.9-fold), ArnD (24.4-fold), ArnA (12.1-fold), ArnB (13.1-fold), and ArnT (5.3-fold), which was a universal mechanism of PB resistance in *K. pneumoniae*, expressed at a high level in PB-resistant strain (Figure 3E). Moreover, the abundance of PhoP-activated palmitoyltransferase PagP and lipid A 1-diphosphate synthase (LpxT) was also elevated in the PB-resistant *K. pneumonia*. These changes suggested that LPS modification is possibly the main pattern of PB resistance in the *K. pneumoniae*.

**FIGURE 4:**
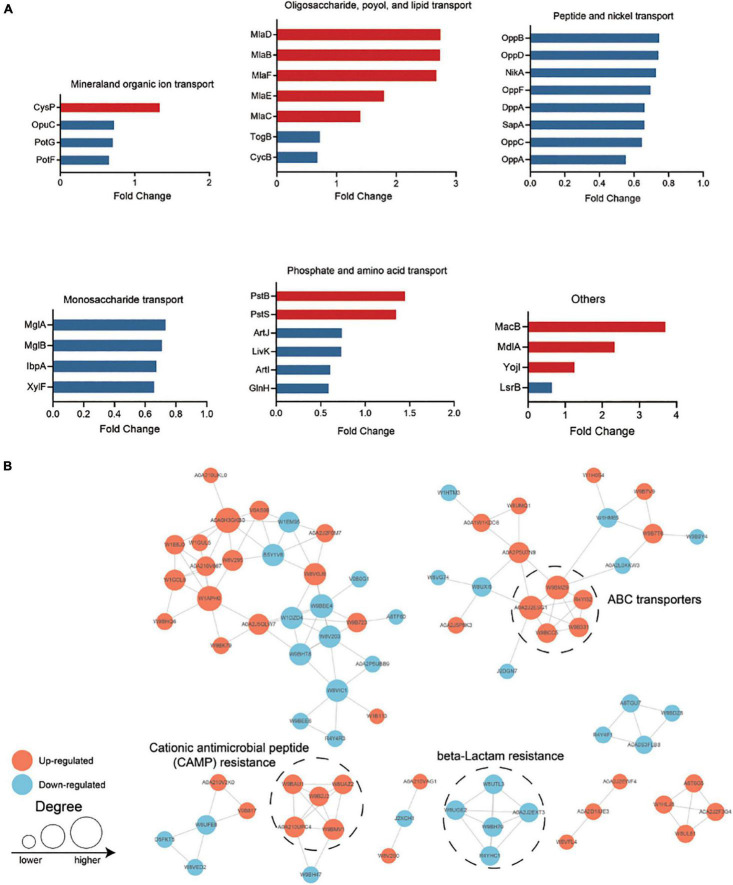
**(A)** Differentially expressed ATP-binding cassette (ABC) transporters in polymyxin B (PB) resistant *Klebsiella pneumoniae*. **(B)** Protein—protein interaction (PPI) network analysis of the remained differentially expressed proteins (DEPs). The size of the node indicates the number of interacting proteins. The upregulated proteins are shown in orange and the downregulated proteins are in blue.

Most ABC transport subunits were inhibited in resistant strain (Figure 4A). Various amino acid and carbohydrate transporters were down-regulated, including lysine/arginine/ornithine (ArgT), glutamine (GlnH), branched amino acid (LivK), maltose (MalE), and D-xylose (XylF), suggesting that PB-resistant *K. pneumoniae* had a decreased ability to utilize carbon and nitrogen sources. The increased ABC transporter subunits were mainly responsible for phosphate (PstS and PstB) and phospholipid transport (MlaFEDB and MlaC). Furthermore, drug efflux pump TolC and MacB were also found to increase in the PB-resistant *K. pneumoniae*.

Among the valine, leucine and isoleucine biosynthesis, expression of acetolactate synthase I/II/III large subunit and acetolactate synthase I/III small subunit were suppressed in the PB-resistant *K. pneumoniae*, but levels of dihydroxy-acid dehydratase, branched-chain amino acid aminotransferase, and threonine dehydratase were elevated.

Central carbon metabolism was partially inhibited, including TCA cycle, pyruvate metabolism and glycolysis/gluconeogenesis. Among these pathways, expression of phosphoenolpyruvate carboxykinase (PckA), malate dehydrogenase (Mqo), acetyl-CoA synthetase (ACS), succinate dehydrogenase iron-sulfur subunit (FrdB), fumarate reductase flavoprotein subunit (FrdA), malate synthase (AceB), malate dehydrogenase (Mdh), 6-phospho-beta-glucosidase (E3.2.1.86A) and glucose-1-phosphatase (Agp) was suppressed in the PB-resistant *K. pneumoniae*, but D-lactate dehydrogenase (LdhA), malate dehydrogenase (ME2), aldose, 1-epimerase(GalM) and fructose-bisphosphate aldolase (FbaB) were activated. In conclusion, changes in metabolism, membrane protein, and transport were important for *K. pneumoniae* under PB pressure.

All the DEPs were submitted to Search Tool for the Retrieval of Interaction Gene/Proteins (STRING) database and Cytoscape software for protein-protein interaction (PPI) network analysis (Figure 4B). Proteins with high confidence score (> 0.7) would be retained and proteins that did not interact with other proteins would be filtered out. The closely interacted DEPs were mainly distributed in three modules, including CAMP resistance pathway, beta-lactam resistance, and ABC transporter.

**FIGURE 5:**
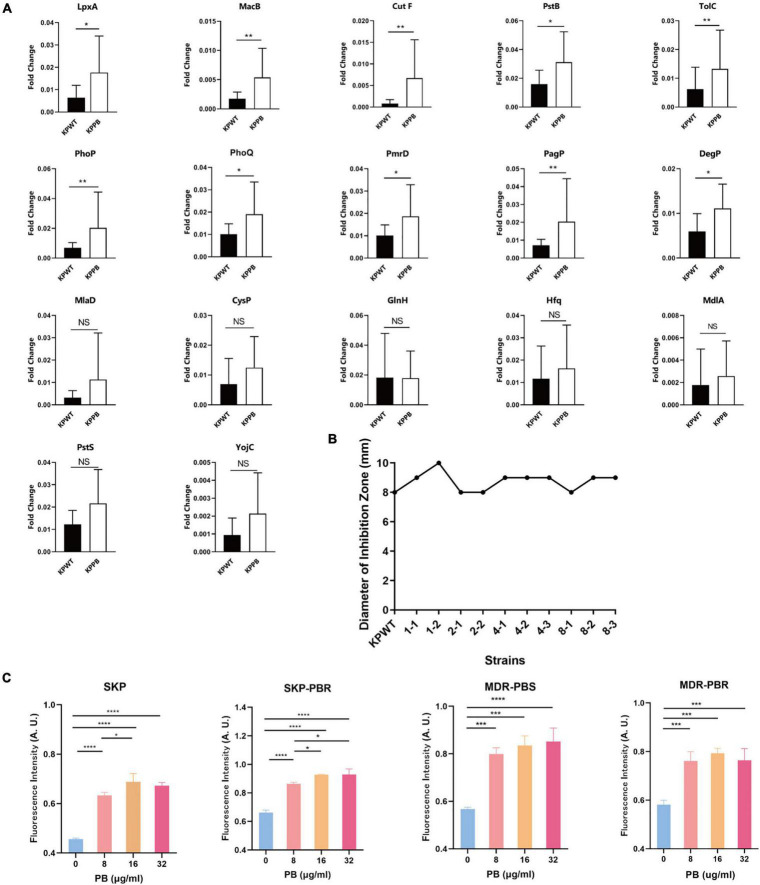
Validation of proteomic results in *Klebsiella pneumoniae*. **(A)** Relative transcript levels of the selected genes in the PB-resistant and susceptible *K. pneumoniae* (*n* = 10). NS, no significance. **(B)** Diameter of inhibition zone of erythromycin disk against *K. pneumoniae*. **(C)** Measurement of ROS production in *K. pneumoniae* clinical isolates treated with PB. SKP, *K. pneumoniae* susceptible to all first-line antibiotics; SKP-PBR, *K. pneumoniae* only resistant to PB; MDR-PBS, clinical isolate only susceptible to PB and tigecycline; MDR-PBR, PB-resistant strain only susceptible to tigecycline. Results are presented as the mean ± standard deviation. *P < 0.05, **P < 0.01, ***P < 0.001, and ****P < 0.0001.

Transcriptional level of the selected DEPs involved in CAMP resistance pathway (*degP*, *cutF*, *lpxA*, *pagP*, *pmrD*, *tolC*, *phoP*, *phoQ*, and *pstB*), ABC transporters (*macB*, *pstS*, *mlaD* and *yojI*) and quorum sensing (*mdlA*, *cysP*, *hfq*, and *glnH*) were tested between PB-resistant and -susceptible *K. pneumoniae*. Results showed that gene expression of all the DEPs was consistent with the proteomic analysis (Figure 5A). Transcriptional level of *lpxA*, *pagP*, *pmrD*, *phoP*, *phoQ, macB*, and *tolC* was significant between the two groups, suggesting that the CAMP resistance pathway may have an impact on PB resistance in *K. pneumoniae*.

In light of that MacB is one of macrolide transporters, K-B method was used to measure the inhibitory zone size of erythromycin disk against KPWT and induced strains (Figure 5B). However, there was no significant change in the inhibition zone diameter of erythromycin disk between the PB-resistant mutants and susceptible strains (with the diameter ranged from 8 to 10 mm).

Since TCA cycle is one of the sources of ROS, in order to investigate whether ROS level is a potential factor related to PB resistance in *K. pneumoniae*, four clinical isolates with different antibiotic susceptibility phenotypes were treated with 0, 8, 16, 32 μg/ml PB for 30 min. Results demonstrated that ROS production of four *K. pneumoniae* clinical isolates increased significantly after exposure to PB (Figure 5C). ROS level caused by 16 μg/ml PB was significantly higher than that caused by 8 μg/ml PB among all strains, but there was no statistical difference in ROS level between 32 μg/ml and 16 μg/ml PB groups.

### Lipid Profile Was Significantly Disturbed in the Polymyxin B-Resistant *Klebsiella pneumoniae*

**FIGURE 6:**
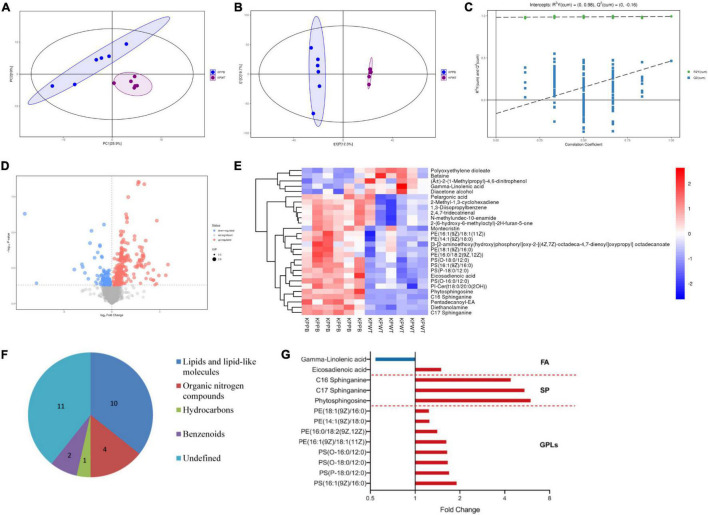
Analysis of the metabolic data. **(A)** Principal component analysis (PCA) score plot. Blue dots represent PB-resistant strains and purple dots represent PB susceptible strains (*n* = 6). **(B)** Orthogonal projection latent structure discrimination analysis (OPLS-DA) score plot. **(C)** OPLS-DA permutation plot. **(D)** Volcano plot shows expression of differential metabolites between PB-resistant and -susceptible *K. pneumoniae*. Diameter of each dot is determined by its VIP value. Upregulated and downregulated metabolites are shown in red and blue, respectively. **(E)** Heatmap shows the expression pattern of differentially expressed metabolites. **(F)** Classification information of the differential metabolites based on HMDB database. **(G)** Fold change of the significantly changed lipids in the PB-resistant *K. pneumoniae*. Red bars represent the lipids with increased abundance in PB-resistant strains, and blue bars represent lipids with decreased abundance. GPLs, glycerophospholipids. SP, sphingolipid. FA, fatty acid.

Quantitative metabolomics was performed to identify key metabolites associated with PB resistance in *K. pneumoniae* (Supplementary Data Sheet 2). The principal component analysis (PCA) score plot showed that samples of each group were clustered tightly and within the 95% confidence interval, indicating that differences in metabolites between the PB-susceptible and -resistant *K. pneumoniae* can be easily distinguished (Figure 6A). OPLS-DA model showed good interpretability (R^2^X = 0.32, R^2^Y = 0.993) and predictability (*Q*^2^ = 0.464) for the samples (Figures 6B,C). Comparative metabolomics analysis had identified 28 differential metabolites, of which 23 were upregulated and 5 were downregulated (VIP > 1 and *P* < 0.05) (Figure 6D).

Expression patterns of differential metabolites between samples were displayed through heatmap (Figure 6E). Details of the differentially expressed metabolites are demonstrated in Table 2. Among the 28 significantly changed metabolites, 10 were lipids and lipid-like molecules, 4 were organic nitrogen compounds, 2 were benzenoids, 1 was hydrocarbons, but classification information of the remaining 11 was not queried in the HMDB database (Figure 6F). Pentadecanoyl-EA and phytosphingosine showed the greatest fold change among all the differential metabolites.

**TABLE 2:** Differentially expressed metabolites between Polymyxin B (PB) resistant and susceptible *Klebsiella pneumoniae.*

SuperClass	MS2 name	type	VIP	*P*-VALUE	Fold Change*^a^*
Lipids and lipid-like molecules	PS(16:1(9Z)/16:0)	Up	2.64	3.2100E-05	1.95
	PE[16:1(9Z)/18:1(11Z)]	Up	1.63	0.0085	1.66
	Eicosadienoic acid	Up	2.46	0.0009	1.53
	PE[16:0/18:2(9Z,12Z)]	Up	2.05	0.0056	1.45
	Pelargonic acid	Up	1.82	0.0321	1.43
	Montecristin	Up	1.93	0.0197	1.43
	PE(14:1(9Z)/18:0)	Up	2.01	0.0064	1.28
	PE(18:1(9Z)/16:0)	Up	1.82	0.0252	1.27
	Polyoxyethylene dioleate	Down	2.06	0.0058	0.62
	Gamma-Linolenic acid	Down	1.87	0.0455	0.53
Organic nitrogen compounds	Phytosphingosine	Up	2.78	0.0004	6.13
	Diethanolamine	Up	2.57	8.9002E-06	1.94
	Diacetone alcohol	Down	1.8	0.0303	0.85
	Betaine	Down	1.88	0.0195	0.65
Benzenoids	1,3-Diisopropylbenzene	Up	2.11	0.0044	1.50
	(Â ±)-2-(1-Methylpropyl)-4,6-dinitrophenol	Down	1.64	0.0329	0.66
Hydrocarbons	2-Methyl-1,3-cyclohexadiene	Up	1.83	0.0394	1.33
Undefined	Pentadecanoyl-EA	Up	2.65	0.0027	6.31
	C17 Sphinganine	Up	2.73	0.0002	5.57
	C16 Sphinganine	Up	2.76	4.1755E-09	4.50
	PS(P-18:0/12:0)	Up	2.59	1.4002E-05	1.73
	PS(O-18:0/12:0)	Up	2.37	0.0008	1.70
	PS(O-16:0/12:0)	Up	2.61	8.4510E-06	1.68
	PI-Cer[t18:0/20:0(2OH)]	Up	2.09	0.0024	1.55
	2-(6-hydroxy-6-methyloctyl)-2H-furan-5-one	Up	1.98	0.0097	1.43
	N-methylundec-10-enamide	Up	1.91	0.0134	1.42
	2,4,7-tridecatrienal	Up	1.97	0.0135	1.41
	[3-[2-aminoethoxy(hydroxy)phosphoryl]oxy-2-[(4Z,7Z)-octadeca-4,7-dienoyl]oxypropyl] octadecenoate	Up	1.79	0.0339	1.28

*^a^Ratio of the relative abundance of metabolites in PB-resistant strains to the PB-susceptible strains.*

It is obvious that the lipid profile was significantly disturbed in the PB-resistant *K. pneumoniae*. Almost all lipids with significant changes were upregulated in PB-resistant strain. The differential metabolites mainly belong to glycerophospholipids, sphingolipids, and fatty acids (Figure 6G). Specifically, levels of phosphatidylserine (PS), phosphatidylethanolamine (PE), phytosphingosine, and sphinganine were significantly elevated. Several fatty acids were perturbed by PB treatment as well, including eicosadienoic acid and γ-linolenic acid. These changes indicate that the function of the cell membrane of *K. pneumoniae* may have changed to resist PB. KEGG enrichment analysis of differential metabolites showed no result (*P* < 0.05).

### Phospholipid Metabolism Was Perturbed and Phospholipid Transport Was Enhanced in Polymyxin B-Resistant *Klebsiella pneumoniae*

**FIGURE 7:**
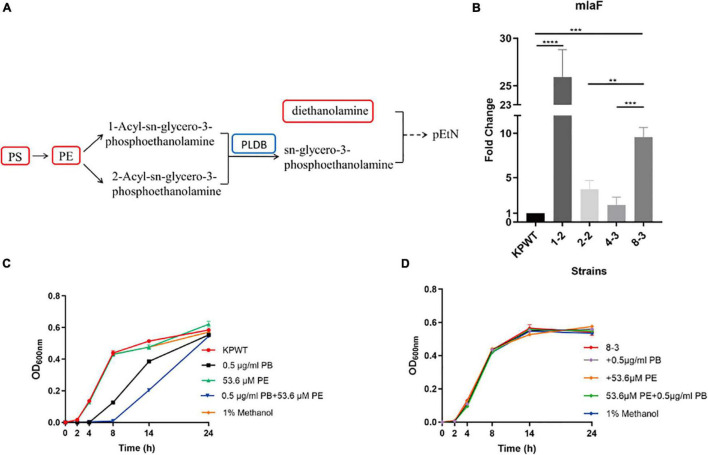
Alterations in phospholipid metabolism and transport in polymyxin B (PB) resistant *Klebsiella pneumoniae*, and effect of phosphatidylethanolamine (PE) on the activity of PB against *K. pneumoniae*. **(A)** Perturbation in glycerophospholipid metabolism. Red box in the flow charts represents the significantly increased metabolites, while blue box indicates the significantly decreased protein. Components without box mean that no differential expression occurs between PB-resistant and -susceptible *K. pneumoniae*. **(B)** Changes in the transcription level of *mlaF* during induction of KPWT. **(C)** and **(D)** Affection of 0.5 μg/ml PB alone or combined with 53.6 μM PE on the growth of KPWT and strain 8-3. Data represent the means ± SD of three independent repeated experiments. **P < 0.01; ***P < 0.001; ****P < 0.0001.

It is notable that specific intermediate metabolites in glycerophospholipid metabolism (PS, PE and diethanolamine) were found to be increased in PB-resistant *K. pneumoniae* (Figure 7A). PE could produce 1-Acyl-sn-glycero-3-phosphoethanolamine under the action of phospholipase A2 (Pla2), or catalyzed by phospholipase A1 (Pla1) to form 2-Acyl-sn-glycero-3-phosphoethanolamine, both of which can synthesize sn-glycero-3- phosphoethanolamine under the action of lysophospholipase (PldB). Accumulation of PS and PE possibly could be attributed to the decreased PldB (0.753-fold) in PB-resistant *K. pneumonia*. Members of Mla pathway by which phospholipids can be transported between the OM and cytoplasmic membrane were upregulated in PB-resistant *K. pneumoniae*.

It was found that transcription levels of *mlaF* were all upregulated in the descendant strains during induction process (Figure 7B). Results suggest that *mlaF* may be a key molecule for *K. pneumoniae* in response to PB stress. However, compared with the group treated by 0.5 μg/ml of PB alone, combination with 53.6 μM PE enhanced bactericidal activity against KPWT within 14 h and regrowth of KPWT was observed after 24 h (Figure 7C). Little discernible bactericidal effect of this combination was observed in strain 8-3 (Figure 7D).

## Discussion

Resistance to PB in the CRKP is a clinical issue worthy of attention. The KPWT clinical isolate used in this study rapidly developed strong and stable resistance under the concentrations of PB ranging from 0.5 × MIC to 4 × MIC. Grown in antibiotic-free medium, the resistant strains induced by 4 μg/ml PB (2 × MIC) were unstable and easily restored their sensitivity to PB. However, the resistance induced by 8 μg/ml (4 × MIC) PB was more stable, and could be maintained after 3-5 passages without antibiotics. The induced PB resistance resulted in almost no fitness cost, with the growth rate of resistant mutants was not significantly different from that of the parent strain. Emergence of such strong and stable resistant strains with little fitness cost poses great difficulties for antimicrobial therapy and a serious threat to public health.

This study elucidated adaptive proteome and metabolome changes of *K. pneumoniae* to PB pressure. Bacterial quorum sensing (QS) is a way of communication between cells, which can be activated by chemical signals outside the cell to regulate biological functions, thereby adapting to the environment (Jiang Q. et al., 2019; Chen et al., 2020). The DEPs involved in QS pathway were all inhibited in PB-resistant *K. pneumoniae*. It is believed that Autoinducer 2 (AI-2) produced by the enzyme LuxS can be taken up by the transporter LsrACDB, and participate in QS signal transduction, which plays a vital role in the formation of biofilms (Xavier and Bassler, 2005; Chen et al., 2020). A study showed that there is a positive correlation between biofilm formation and carbapenem resistance in clinical *K. pneumoniae* strains, and the ability of biofilm formation was significantly related to the clinical outcome of patients (Devanga Ragupathi et al., 2020). Proteins involved in the quorum sensing pathway were found to be inhibited in PB-resistant strains. Contrary to proteomics results, enhanced biofilm formation ability was observed in the induced strains. It has been reported that *K. pneumoniae* in biofilms showed higher resistance to polymyxin than the planktonic cells (Naparstek et al., 2014). This difference between protein level and phenotype may be caused by post-translational modifications of proteins (Lassak et al., 2019). Defects in *K. pneumoniae* porins OmpK35 and OmpK36 (their *E. coli* homologs are OmpF and OmpC, respectively) could lead to reduced sensitivity to carbapenem (Sugawara et al., 2016; Hamzaoui et al., 2018). In this study, proteomics data showed that expression levels of LsrB, OmpK35 and OmpK36 were decreased in PB-resistant strain. These changes may explain why the MIC value of PB-resistant *K. pneumoniae* to IPM and MEM had decreased (but still maintained resistance). The significantly decreased host factor-I protein (Hfq) participates in the QS pathway and is an RNA chaperone that can promote the pairing of small RNAs (sRNAs) with target mRNA, mediating the regulation of mRNA stability and translation (Kavita et al., 2018). This regulation helps bacteria adapt during stress. However, there was no significant difference in the transcriptional level of Hfq between PB-resistant and -susceptible *K. pneumoniae*, which may also be contributed to post-transcriptional modification.

Polymyxin can stimulate the accumulation of ROS and cause rapid killing of bacteria (Yu et al., 2017; Ajiboye et al., 2018). This effect has been proved in *Bacillus subtilis* and *Bacillus polymyxa* (Yu Z. et al., 2019). When the concentration of ROS reaches a certain level, it can cause oxidative damage to bacterial DNA, protein, and lipids, leading to bacterial death (Yu et al., 2017; Liu J. et al., 2020). Cellular respiration dependent on TCA cycle is an important source of antibiotic induced oxidative stress (Kohanski et al., 2007). After exposure to antibiotics, a moderate increase in the consumption of NADH produced by NAD^+^ through the TCA cycle may induce production of O_2_^–^ by accelerating the respiratory chain (Lobritz et al., 2015). Cytochrome o ubiquinol oxidase subunit II (CyoA), FrdA, FrdB, Mdh, and Mqo were involved in the TCA cycle and/or oxidative phosphorylation and were decreased in PB-resistant *K. pneumoniae*. Moreover, study has indicated that PB is capable of promoting ROS levels via activating α-ketoglutarate dehydrogenase and Mdh in Gram-positive bacteria, thus playing an antibacterial role (Yu Z. et al., 2019). Our study confirmed that high concentrations of PB can stimulate ROS production in both antibiotic susceptible *K. pneumoniae* and multidrug-resistant *K. pneumoniae*, suggesting that down-regulation of ROS production may be a potential mechanism of PB resistance in *K. pneumoniae*. There was no significant difference in ROS levels caused by 16 μg/ml and 32 μg/ml PB, suggesting that bacterial stress response may reach its limit at this concentration. Moreover, inhibition of central carbon metabolism in PB-resistant strain was also manifested in pyruvate metabolism. Previous study obtained PB-resistant *K. pneumoniae* strains using a similar approach to our study and performed transcriptome sequencing, which found that TCA cycle was inhibited and *K. pneumoniae* shifts to fermentative growth upon PB pressure (Ramos et al., 2016). Similar to our results, it was found that transcription levels of Frd and CyoA were down-regulated in the induced resistant strains. Taken together, metabolic shifts may be associated with oxidative stress and PB resistance in *K. pneumoniae*. A number of studies have reported that changes in central carbon metabolism were related to the acquisition of antibiotic resistance in bacteria (Peng et al., 2015; Cheng et al., 2018; Kawai et al., 2019; Yu W. B. et al., 2019; Zhang et al., 2020). Reduction of TCA cycle led to decreased ROS production, thereby weakening the killing effect of antibiotics on bacteria (Zhang et al., 2020). This situation can be reversed by exogenous glucose. It shows that *K. pneumoniae* may protect itself from oxidative attack by regulating the production of ROS under PB pressure, which helps bacterial survival.

The ABC transporter catalyzes transport reactions, including uptake of micronutrients into bacteria (Locher, 2016). Reduced ABC transporter in the PB-resistant *K. pneumoniae* was responsible for absorption of amino acids and carbohydrates. The reduced nutrient intake might limit bacteria growth (Pal et al., 2019). Protein and transcriptional levels of the efflux pumps TolC and MacB in PB-resistant *K. pneumoniae* were significantly increased. The efflux pump MacAB was first discovered in *Escherichia coli* (*E. coli*) and proved to be link to macrolide resistance (Kobayashi et al., 2001). Nevertheless, the diameter of the inhibition zone of erythromycin disk against the induced strains only slightly altered in comparison with the parent strain. This may be due to the strong resistance of KPWT strain to erythromycin (with diameter of inhibition zone = 8 mm). Although MacAB did not contribute to erythromycin resistance in *Serratia marcescens*, it affected biofilm formation ability and was found to protect bacteria from PB. Furthermore, MacAB was essential for survival of *S. marcescens* during oxidative stress (Shirshikova et al., 2021).

Branched-chain amino acids (BCAA, including leucine, isoleucine, and valine) are an important class of nutrients and necessary for the synthesis of protein and membrane branched-chain fatty acids, which are important for bacterial membrane stability and environmental adaptation (Kaiser et al., 2018). *Staphylococcus aureus* grown on medium lacking leucine or valine exhibited a degree of growth lag. In the PB-resistant *K. pneumoniae*, expression of various enzymes involved in the valine, leucine and isoleucine biosynthesis was disturbed. But growth of the resistant strains in our study did not show significant defect.

Response of bacteria to antimicrobial is multifactorial (De Majumdar et al., 2015). Perturbation of the permeability barrier is considered a key step in the formation and emergence of higher levels of resistance (Vaara, 1993; Jiang J. H. et al., 2019; Sheng et al., 2019; Zgurskaya and Rybenkov, 2020). It is widely approved that PB destroys cell membrane by combining with LPS of Gram-negative bacteria, causing osmotic imbalance, leading to cell death (Han et al., 2019b). Consistent with this, down-regulation of the osmoprotectant betaine and its transporter subunit OpuC were identified in PB-resistant *K. pneumoniae* (Nau-Wagner et al., 1999; Guillot et al., 2000). Alterations of LPS help *K. pneumoniae* survive under polymyxin (De Majumdar et al., 2015; Aye et al., 2020). Proteomic results indicated that LPS modification is still the main mechanism of PB resistance in *K. pneumoniae*. In the CAMP resistance pathway, PhoP/PhoQ, PhoP-activated ArnBCAD, and PagP were highly expressed in PB-resistant strain. Increased ArnBCAD mediates the addition of L-Ara4N to lipid A, reducing the negative charge carried by LPS and the ability of bacteria to bind to PB (Cannatelli et al., 2013; Aye et al., 2020). In Gram-negative bacteria, PagP could use PE as an acyl donor to transfer a palmitoyl (C16:0) group to lipid A, achieving hepta-acylation (Bishop et al., 2000; Boll et al., 2015). The significantly upregulated PE in PB-resistant *K. pneumoniae* may contribute to hepta-acylation of lipid A. Formation of protective hepta-acylated lipid A can strengthen the LPS of the OM barrier and reduce host immune recognition (Boll et al., 2015). C12/C14 acylated lipid A (hepta-acylated lipid A) produced by LpxL/LpxM in *Acinetobacter baumannii* also conferred resistance to polymyxin (Boll et al., 2015). LpxT adds a phosphate to the 1-position of lipid A to produce a 1-diphosphate species (1-PP), which increases the negative charge on the cell surface. Upregulation of LpxT was detected in PB-resistant *K. pneumoniae* (Touze et al., 2008). Theoretically, this change is not conducive to the resistance of bacteria to PB. It is speculated that this effect may be offset by other LPS modifications.

The key role of the OM in Gram-negative bacteria is to act as a permeation barrier to protect the bacteria from the host and the environment (Hancock, 1997; Liu J. et al., 2020). Bacteria can adjust the fluidity of the cell membrane by adjusting the types and composition of fatty acids and phospholipids, thereby maintaining the stability and normal physiological functions of the cell membrane, and adapting to osmotic stress (Jia et al., 2017). Changes in eicosadienoic acid and γ-linolenic acid were found in PB-resistant *K. pneumoniae*, which may be adaptive changes to PB stress. Lipids were the most important metabolic changes in PB-resistant *K. pneumoniae*, especially the increased PS and PE. Accumulation of PS and PE may be caused by the reduced abundance of PldB. Accumulated phospholipids have been observed in several polymyxin-resistant Gram-negative bacteria, including *E. coli*, *K. pneumoniae*, and *Pseudomonas aeruginosa* (Jasim et al., 2018; Han et al., 2019b; Li et al., 2019; Aye et al., 2020). Phospholipids (including glycerophospholipids and sphingolipids) and fatty acids are important components of cell membrane and essential for OM integrity and bacterial survival (Sohlenkamp and Geiger, 2016). Upregulation of the asymmetry-maintenance system Mla (MlaFEDB and MlaC) was observed in PB-resistant *K. pneumoniae*. It is believed that the Mla system is responsible for the transport of phospholipids between the inner membrane and OM (Hughes et al., 2019). In ΔmlaE/ΔpldA double mutant *Acinetobacter baumannii*, MIC value of PB has also changed compared with its wild type (Powers and Trent, 2018). We did find that transcription level of *mlaF* in *K. pneumoniae* was increased after exposure to PB, suggesting that Mla pathway may play a role in bacterial survival under PB stress. Contrary to expectation, adding exogenous PE to the medium inhibited the growth of bacteria even more rather than protecting *K. pneumoniae* from PB. Perhaps there were multiple complex factors that affect the interaction between PB, PE, and bacteria, such as the treated concentration of PE (PE concentrations in this study were referenced from a previous study on the enhanced antibacterial effect of PB on *Salmonella* by lysophosphatidylcholine) (Yadav et al., 2020) or the function of endogenous and exogenous PE on bacteria may be different, etc. It should be more accurate to study effect of PE on PB resistance by interfering endogenous PE synthesis pathway in *K. pneumoniae*.

**FIGURE 8:**
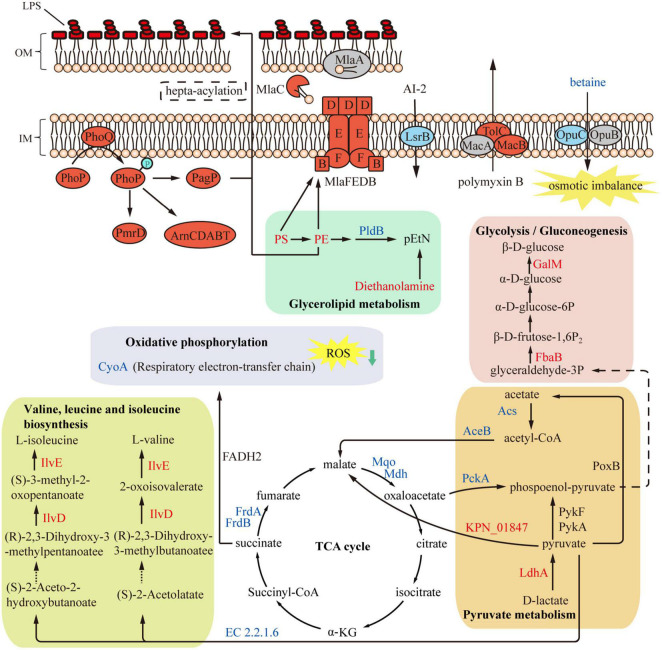
Overview of metabolome and proteome differences between polymyxin B (PB) resistant and susceptible *Klebsiella pneumoniae*. Expression of several LPS-modifying enzymes was upregulated in PB-resistant *K. pneumoniae*, while the TCA cycle was inhibited and thereby production of ROS was reduced. Accumulated PS and PE in the glycerophospholipid metabolism were transported to the cell membrane through Mla system to strengthen the cell membrane. Red and blue represent upregulated and decreased proteins or metabolites (except LPS). Gray or black indicates proteins or metabolites with insignificant changes in expression.

In conclusion, this study provides insights for understanding the resistance mechanism of PB in *K. pneumoniae* (Figure 8). Results of proteomics and metabolomics analyses emphasize the role of central carbon metabolism and cell membrane remodeling in the development of PB resistance.

## Materials and Methods

### Bacterial Strains and Reagents

The *K. pneumoniae* clinical isolates used in this study were obtained from the gastric juice, sputum and feces of neonate and adult inpatients in the Second Xiangya Hospital of Central South University (Changsha, Hunan, China). All isolates were identified by Matrix-Assisted Laser Desorption/Ionization Time of Flight Mass Spectrometry (Bruker Daltonik, Bremen, Germany). PB solution was prepared immediately before each experiment with sterile distilled water and PB powder (Sangon Biotech, Shanghai, Co., Ltd., Shanghai, China). Phosphatidylethanolamine (Sigma-Aldrich, Melbourne, VIC, Australia) was dissolved in methanol as recommended to prepare a stock solution with a concentration of 53.6 μmol/ml. The final concentration of phosphatidylethanolamine used to treat bacteria was based on previous literature (Yadav et al., 2020).

### Antimicrobial Susceptibility Test

Antimicrobial susceptibility test was performed on the last generation of *K. pneumoniae* strains passaged in each concentration using Phoenix 100 automatic microbial analysis system (Becton Dickinson, Franklin Lakes, NJ, United States). The MIC values of *K. pneumoniae* isolates and induced strains to polymyxin B were further confirmed using broth dilution method in Mueller-Hinton (MH) broth (Rishui Biotech Co., Ltd., Qingdao, China) recommended by the Clinical and Laboratory Standards Institute (CLSI) guidelines (Institute, 2006). The induced resistant strains were plated on antibiotic-free blood agar at 37°C for 18–24 h. Single colony was selected and inoculated to blood ager plate for subculture. Resistance phenotype of *K. pneumoniae* against erythromycin was obtained by K-B method. Erythromycin disk was purchased from Oxoid (United Kingdom). Since CLSI does not provide breakpoint of erythromycin against *Enterobacteriaceae*, the inhibition zone diameter was just recorded. Results of antimicrobial susceptibility tests were interpreted using breakpoints recommended by the CLSI.

### *In vitro* Induction of Polymyxin B Resistance

A single colony of *K. pneumoniae* clinical isolates was inoculated into Luria-Bertani (LB) broth (Sangon Biotech, Shanghai, Co., Ltd., Shanghai, China) and grew at 37°C 180 rpm. Bacterial suspension was serially passaged in LB broth with increasing concentrations of PB (1, 2, 4, and 8 μg/ml) to induce resistance. Briefly, the overnight culture was diluted 1:100 in LB broth with PB. Since the MIC of KPWT to PB was 2 μg/ml, only two generations were passaged in LB broth with PB concentration of 1 μg/ml and 2 μg/ml, and three successive generations were passaged in liquid medium with PB concentration of 4 μg/ml and 8 μg/ml.

### Growth Curve Measurement

Bacterial cells were inoculated into 5 ml fresh LB broth to reach a bacterial density of about 1 × 10^6^ CFU/ml. PB and/or PE were added to the tubes to achieve the desired concentration. The cultures were grown at 37°C and 180 rpm. For the PE treated group, bacterial suspension containing 1% methanol was used as control. Optical density at 600 nm (OD_600nm_) of each culture at different time points (0, 2, 4, 8, 14, 24 h) was measured by Thermo Multiskan GO (ThermoFisher Scientific, Waltham, MA, United States) to monitor bacterial growth condition. OD value of each testing group minus that of MH broth without bacteria. The results represented the mean value of three independent replicates.

### Biofilm Formation Assay

Biofilm formation was determined by crystal violet staining method as described in previous literature (Vuotto et al., 2017; Fang et al., 2021). For each strain, 200 μl bacterial suspension (1 × 10^6^ CFU/ml) was inoculated to three wells of 96-well plates (Corning, Corning, NY, United States), sterile MH broth served as negative control. After static incubation at 37°C for 24 h, each well was washed with sterile normal saline for three times to remove the planktonic cells. After drying, biofilms were stained using 0.1% (wt/vol) crystal violet for 15 min and washed with sterile normal saline three times. Decolorization was performed with 95% ethanol (v/v) and the absorbance at 595 nm was measured by Thermo Multiskan GO (ThermoFisher Scientific, Waltham, MA, United States). The experiment was performed in triple and results showed mean ± SD.

### Protein Extraction and Digestion

Three individual colonies of KPWT and KPPB were collected and rapidly frozen using liquid nitrogen. After addition of lysis buffer (8 M urea, 1% Protease Inhibitor Cocktail), samples were sonicated three times. Subsequently, the supernatant was collected by centrifugation at 12,000 × *g* for 10 min at 4°C. The protein concentration was determined using bicinchoninic acid (BCA) kit (Beyotime, Shanghai, China).

A total of 5 mM dithiothreitol (DTT) was added to the protein preparation. After incubation at 56°C for 30 min, 11 mM iodoacetamide was added into the protein solution for 15 min in the dark at room temperature. 100 mM tetraethylammonium bromide (TEAB) was used to dilute the protein sample and make the urea concentration less than 2M. Finally, the protein sample was digested by trypsin at a ratio of 1:50 (overnight) and 1:100 (4 h).

### Tandem Mass Tag Labeling, High-Performance Liquid Chromatography Fractionation and LC-MS/MS Analysis

The digested peptide was desalted using Strata X C18 SPE column (Phenomenex, United States) and vacuum-dried. Next, peptide was reconstituted in 0.5 M TEAB and labeled with tandem mass tag (TMT) kit (Thermo Fisher Scientific, Waltham, MA, United States). Labeling condition was processed according to the manufacturer’s protocol.

The TMT-labeled peptide was fractionated by high pH reverse-phase HPLC with Agilent 300Extend C18 column (5 μm particles, 4.6 mm ID, 250 mm length). Briefly, peptides were first separated into 60 fractions over 60 min with a gradient of 8% to 32% acetonitrile (pH 9.0). Then the peptides were combined into 18 components and dried by vacuum centrifugation.

Solvent A (0.1% formic) was used to dissolve the tryptic peptides, and then was separated by EASY-nLC 1000 (Thermo) at 400 nL/min with a homemade reversed-phase analytical column (15-cm length, 75 μm i.d.). Gradient solvent B (0.1% formic acid dissolved in 90% acetonitrile) was used in increasing steps from 8% to 23% (26 min) to 23% to 35% (8 min) and climbing to 80% in 3 min, then held at 80% for the last 3 min.

After liquid chromatography (LC) separation, the peptides were then injected into nanospray ion (NSI) source followed by tandem mass spectrometry (MS/MS) in Q ExactiveTM Plus (Thermo) with the UPLC. The electrospray voltage was 2.0 kV and m/z scan range were 350 to 1800 for full scan. Peptide fragments were detected using the Orbitrap. The resolution was 17,500. 20 MS/MS scans were performed after one MS scan with 15.0s dynamic exclusion. Automatic gain control (AGC) was 5E4 with fixed first mass set as 100 m/z.

### Data Processing

The obtained MS/MS data were processed by the Maxquant search engine (v.1.5.2.8). Uniprot *Klebsiella pneumoniae* database (171,340 sequences) linked to the reverse decoy database was used to search for tandem mass spectra. An anti-database was added to calculate the false-discovery rate (FDR), and a common contamination library was added to the database to eliminate the influence of contaminating proteins among the identification results. The remaining parameters were as follows: set trypsin/P as a cleavage enzyme and allowed up to 2 missing cleavages; the mass tolerance of the primary precursor ions of First search and Main search was set to 20 ppm and 5 ppm; the mass tolerance for the secondary fragment ions was 0.02 Da. The quantitative method was TMT-6plex. The FDR for protein and PSM identification were set at 1%.

### Measurement of Reactive Oxygen Species Generation

Reactive oxygen species (ROS) levels in *K. pneumoniae* treated by PB was measured with 2′,7′-dichlorofluorescein diacetate (DCFH-DA), following the manufacturer’s instruction (MedChemExpress, Monmouth Junction, NJ, United States). Strains were incubated overnight at 37°C with shaking at 180 rpm. The culture was washed three times with phosphate buffered solution (PBS) and resuspended, adjust OD_600_ = 0.5. DCFH-DA was added to bacterial suspension to make a final concentration of 10 μM. The samples were incubated at 37° for 30 min and then washed with PBS three times. PB was added to make the final concentration of 0, 8, 16, 32 μg/ml. After 30 min of treatment, the fluorescence intensity was detected immediately with the excitation wavelength at 488 nm and the emission wavelength at 525nm using the Varioskan Lux Microplate reader (ThermoFisher Scientific, Waltham, MA, United States). This experiment was repeated three times.

### Preparation of Metabolomics Samples and LC-MS/MS Analysis of Cellular Metabolites

The cell culture (mid-log growth phase) was selected and mixed with normal saline until the optical density at 600 nm (OD_600_) reaches 0.50 ± 0.02 to ensure the total amount of bacteria cells is 1 × 10^8^CFU/ml. 100 μl of the bacteria liquid was diluted ten times in a 1 ml EP tube to ensure bacteria amount was 1 × 10^7^ cells. Cell pellets were collected from the 1ml culture after centrifugation at 1,000 × *g* and 4°C for 10 min. The supernatant was removed and bacteria was quickly quenched in liquid nitrogen for 30 s to stop the metabolic process. After quenching, the bacteria were thawed on ice, washed twice with 4°C or 20°C precooled 1 × phosphate-buffered saline (PBS) to remove the remaining medium components, and then was centrifuged (10 min at 1,000 × *g*, 4°C) to remove the PBS buffer. Six biological replicates were prepared for PB susceptible and -resistant strains, respectively. The samples were stored at –80°C before LC-MS/MS analysis.

Cellular metabolites of the PB-resistant and -susceptible *K. pneumoniae* were extracted through a previously reported method (Cai et al., 2015). 600 μL extraction solution (methanol: acetonitrile: water = 2:2:1 (V/V), containing isotopically labeled internal standard mixture) was added to the samples, and bacteria samples were transferred into EP tube. After vortexing for 30s, the solution was homogenized at 35 Hz for 4 min and sonicated for 5 min (in ice water). This step was repeated for 3 times and then samples were incubated for 1 h at –40°C. Subsequently, the supernatant was collected by centrifugation at 12,000 rpm for 15 min at 4°C and transferred to a clean glass vial for LC-MS/MS analysis. The same amount of supernatant from all samples was mixed together as a quality control (QC) sample.

Detection of metabolites was performed using an ultra high-performance liquid chromatography (UHPLC) system (Vanquish, ThermoFisher Scientific, Waltham, MA, United States) with a UPLC BEH Amide column (2.1 mm × 100 mm, 1.7 μm, Waters) coupled to Q Exactive HFX mass spectrometer (Orbitrap MS, ThermoFisher Scientific, Waltham, MA, United States) in both positive and negative ion modes. The UHPLC-QTOF/MS method has been described in detail in the previous literature (Wang et al., 2016). Mobile phase A was composed of 25 mmol/L ammonium acetate and 25 mmol/L ammonia hydroxide in water (pH = 9.75), and mobile phase B was acetonitrile. Gradient conditions were as follows: 0∼0.5 min, 95%B; 0.5∼7.0 min, 95%∼65% B; 7.0∼8.0 min, 65%∼40% B; 8.0∼9.0 min, 40% B; 9.0∼9.1 min, 40%∼95% B; 9.1∼12.0 min, 95% B. Mobile phase flow rate was 0.5 mL/min, the injection volume was 2 μl, and the column temperature and auto-sampler temperature was 25 and 4°C, respectively.

Under the control of the acquisition software (Xcalibur, ThermoFisher Scientific, Waltham, MA, United States), the QE HFX mass spectrometer was used to collect MS/MS spectra on information-dependent acquisition (IDA) mode. The ESI source conditions were as follows: Aux gas flow rate,10 Arb; sheath gas flow rate, 50 Arb; capillary temperature, 320°C; full MS resolution, 60000; MS/MS resolution, 7500; collision energy, 10/30/60 in NCE mode; spray Voltage, 3.5 kV (positive) or –3.2 kV (negative), respectively.

### Data Preprocessing and Statistical Analysis

The raw data were converted to mzXML format by ProteoWizard software and analyzed with R package (based on XCMS) including peak detection, extraction, alignment, and integration. An in-house MS2 database (Biotree Biotech. Co., Ltd., Shanghai, China) was then applied in metabolite annotation with the cutoff set at 0.3. The measurements were filtered if detected peaks were less than half of QC samples. The final data set contained information including the peak number, sample name, and normalized peak area. Then the data were submitted to the SIMCA15.0.2 software package (Sartorius Stedim Data Analytics AB, Umea, Sweden) for Principal component analysis (PCA) and supervised orthogonal projection latent structure discrimination analysis (OPLS-DA) to visualize global metabolic differences between the PB susceptible and –resistant *K. pneumoniae*. To minimize the impact of noise and high variance of variables, data was scaled and logarithmically transformed before analysis. The variable importance in the projection (VIP) of the first principal component in OPLS-DA analysis and *P*-value (Student’s *t*-test) were used as the threshold to identify the significantly changed metabolites (VIP > 1 and *p* < 0.05).

### Bioinformatic Analysis

Gene Ontology (GO) annotation and COG analysis of the DEPs were performed through UniProt-GOA database (www^1^) and Evolutionary Genealogy of Genes: Non-supervised Orthologous Groups (EggNOG^2^) database, respectively. InterProScan software based on the corresponding InterPro domain database^3^ was used to annotate protein domain of the DEPs. KEGG pathway annotation was obtained through KEGG online service tool KAAS and KEGG mapper. The subcellular localization of the DEPs was predicted using WoLF PSORT: protein localization predictor.

Enrichment analyses for GO, KEGG pathway, and protein domains were performed separately. Entries with *P-*values < 0.05 were considered significant.

The PPI network of the DEPs was obtained in the STRING database, and proteins with confidence score > 0.7 (high confidence) would be retained. Cytoscape software was used to visualize the PPI network.

The KEGG database^4^ and human metabolome database (HMDB^5^) were used to annotate the metabolites. Pathway enrichment analysis of the significantly changed metabolites was performed using the KEGG database and MetaboAnalyst^6^.

### Reverse Transcription Quantitative PCR

Transcriptional levels of 19 DEPs (*degP, cutF, lpxA, pagP, pmrD, tolC, pstB, phoP, phoQ, yojI, macB, pstS, mdlA, mlaD, cysP, hfq*, and *glnH*) were verified by reverse transcription quantitative PCR (RT-qPCR). Total RNA of the isolates was extracted using the RNAprep pure Cell/Bacteria Kit (Tian Gen Biotech, Beijing, Co., Ltd., Beijing, China).

The extracted RNA was reverse transcribed into cDNA using Transcriptor cDNA Synth. Kit 2 (Roche, Switzerland). The reaction conditions were as follows: 55°C for 30 min, 25°C for 10 min, and 55°C for 30 min. The cDNA was then amplified by SYBR qPCR Mix Kit (Sangon Biotech, Shanghai, Co., Ltd., Shanghai, China) according to the following procedures: 40 circles of 95°C for 5 min, 95°C for 10 s, 55°C for 15 s, and 72°C for 15 s. The primer sequences are given in Supplementary Table 1.

The 16S RNA was used as an internal standard and was used to normalize the expression level of target genes. Relative expression of these selected genes was quantified by the 2^–ΔΔCT^ method. Statistical analyses were performed by SPSS 22.0 and GraphPad 7.0. These data were presented as mean ± standard deviation. The differences were assessed by the Wilcoxon Signed Rank Test. *P* < 0.05 was considered statistically significant.

### Statistical Analysis

Statistical analysis was performed using GraphPad Prism 6. All data were demonstrated as mean ± SD. Unless otherwise stated, *P*-values were calculated by unpaired *t*-test between two groups or one-way ANOVA between multiple groups (*P < 0.05, ^**^P < 0.01, ^***^P < 0.001, and ^****^P < 0.0001).

## Data Availability Statement

The proteomics data presented in the study are deposited in the ProteomeXchange Consortium via the PRIDE (Perez-Riverol et al., 2019; Deutsch et al., 2020) partner repository, accession number PXD030940. The metabolomics data presented in the study are deposited in the EMBL-EBI MetaboLights repository (Haug et al., 2020), accession number MTBLS4132.

## Author Contributions

JT and MW designed the experiments. JT, XC, and CL performed the experiments. JT and XC analyzed the data and wrote the manuscript. XW, MW, and XL revised the manuscript. All authors read and approved the final manuscript.

## Conflict of Interest

The authors declare that the research was conducted in the absence of any commercial or financial relationships that could be construed as a potential conflict of interest.

## Publisher’s Note

All claims expressed in this article are solely those of the authors and do not necessarily represent those of their affiliated organizations, or those of the publisher, the editors and the reviewers. Any product that may be evaluated in this article, or claim that may be made by its manufacturer, is not guaranteed or endorsed by the publisher.

## Footnotes

1
http://www.ebi.ac.uk/GOA/

2
http://eggnog5.embl.de/#/app/home

3
http://www.ebi.ac.uk/interpro/

4
http://www.genome.jp/kegg/

5
http://www.hmdb.ca

6
http://www.metaboanalyst.ca/
