# Supplementary material for: Cell Membrane Remodeling Mediates Polymyxin B Resistance in Klebsiella pneumoniae: An Integrated Proteomics and Metabolomics Study
Source: Front Microbiol. 2022 Feb 10;13:810403. doi: 10.3389/fmicb.2022.810403 (PMC8866958; doi:10.3389/fmicb.2022.810403)
Supplement: Supplementary file 1 [file Table_1.docx]

## Supplementary Table

**Supplementary Table 1.** Primer sequences of genes used in RT-qPCR

| Gene | Primer sequence (5′-3′) | Protein accession |
| --- | --- | --- |
| *nlpE* | sense: 5'-AACAA TGCCCAGCTTGAACG-3'  anti: 5'-CGGGTTCGCTTCGAGAGTAA-3' | A0A210VJ88 |
| *cysP* | sense: 5'-GCTCCTA TGA TGTCTCCCGC-3'  anti: 5'-A TGCGACTGCTTGA TGGTCA-3' | W9BAC9 |
| *degP* | sense: 5'-GGCACCGAACTGAACTCTGA-3'  anti: 5'-A TTACCTGGCTGACGAAGGC-3' | A0A0H3GJM8 |
| *glnH* | sense: 5'-CTCGACTACACCCTGAAGCC-3'  anti: 5'-TACGCTCGTCGGTAA TGGTG-3' | W9BFE7 |
| *hfq* | sense: 5'-GCTGCAAGGGCAAA TTGAGT-3'  anti: 5'-TAGACCA TCTGGCTGACCGT-3' | W8UPJ3 |
| *lpxA* | sense: 5'-A TTCA TCGCGGCACAGTACA-3'  anti: 5'-GA TCGCCAAGCGTACAA TCG-3' | A0A2J5QND2 |
| *macB* | sense: 5'-GCGTAGCA TTGTCGCTGA TG-3'  anti: 5'-GGTCAGCGTCGAACAGAGAA-3' | A0A2P5U7N9 |
| *mdlA* | sense: 5'-GACGCAACAGCA TTACACCG-3'  anti: 5'-ACA TAGCGCAGCAGGTAGAC-3' | A0A210VH99 |
| *mlaD* | sense: 5'-GGA TACCAGTTCGTTGGCGA-3'  anti: 5'-AGGA TAGTCGTCCCGAGGTC-3' | W9B331 |
| *pagP* | sense: 5'-CACCTCGGCCTTGGCTA TAC-3'  anti: 5'-TGCCGGACCGTAACCTA TTG-3' | W1HU43 |
| *pmrD* | sense: 5'-AGA TTGAAGCCTGTCGCCTC-3'  anti: 5'-TAA TGAGTGGCGTTGCGGA T-3' | A0A0G3RTK6 |
| *pstB* | sense: 5'-TGGTGAA TACAGCTCCGGGT-3'  anti: 5'-ATAAACGCCGTCACCTGGTT-3' | W1HXM2 |
| *pstS* | sense: 5'-CTCTGGTGGCGTTAAGCAGA-3'  anti: 5'-GTGGGGAACTGGAACAGACC-3' | W8V7K8 |
| *tolC* | sense: 5'-CACAA TACGA TGCCGTGCTG-3'  anti: 5'-AGCCA TTCACGTTCAGGGAG-3' | A0A1W1KDD8 |
| *KPNJ2_04787* | sense: 5'-CTA TGTTGAAGCGAACGGCG-3'  anti: 5'-TCAA TCAGGGTGTGGTTCGG-3' | W8V0W8 |
| *yojI* | sense: 5'-CAAACA TA TGGCCAGCCTGC-3'  anti: 5'-CGGTTGAGGGTCAGCTCTTT-3' | W1ANY7 |
| *phoP* | sense:5'-GGAGA TGTTCGCCCAGA TAG-3'  anti: 5'-GTTGAGGA TAA TGCCCTGCT-3' | W1HX46 |
| *phoQ* | sense: 5'-GCGTA TGGAAGGTCTGGTGA-3'  anti: 5'-CGTCGTTTTCTCCTGGCTGA-3' | W1DMH1 |
| *mlaF* | sense: 5'-CGCGTATATCGTGGCGGATA-3'  anti: 5'-ATGGTGATAGTCGCCAGCAG-3 | W9BCC5 |
| *16S RNA* | sense: 5'-GGAGGAAGGTGGGA TGACG-3'  anti: 5'-A TGGTGTGACGGGCGGTGTG-3' | - |
